# Postoperative complications and unanticipated healthcare encounters following mini-laparotomy vs. laparoscopic/robotic-assisted sacrocolpopexy: a comparative retrospective study

**DOI:** 10.1186/s12905-024-03011-4

**Published:** 2024-03-13

**Authors:** Henry H. Chill, Alireza Hadizadeh, Claudia Paya-Ten, Angela Leffelman, Cecilia Chang, Nani P. Moss, Roger P. Goldberg

**Affiliations:** 1 Division of Urogynecology, University of Chicago Pritzker School of Medicine, NorthShore University HealthSystem, Skokie, IL USA; 2 Department of Obstetrics and Gynecology, Hadassah Medical Center, Faculty of Medicine, Hebrew University of Jerusalem, Jerusalem, Israel; 3 NorthShore University HealthSystem Research Institute, Evanston, IL USA; 4 Division of Urogynecology, Trinity Health of New England Medical Group, Bloomfield, CT USA

**Keywords:** Pelvic organ prolapse, Mini-laparotomy, Apical prolapse, Vaginal vault prolapse, Sacrocolpopexy, Laparoscopic surgery, Robotic-assisted laparoscopic surgery

## Abstract

**Background:**

Pelvic organ prolapse is a debilitating condition impacting lives of millions of women worldwide. Sacrocolpopexy (SCP) is considered an effective and durable surgical technique for treatment of apical prolapse. The aim of this study was to compare short-term outcomes including postoperative complications and unanticipated healthcare encounters between patients who underwent SCP with a mini-laparotomy approach compared to patients treated with laparoscopic and robotic-assisted laparoscopic SCP.

**Methods:**

This was a retrospective cohort study including patients treated for apical prolapse at a university affiliated urogynecology practice. Patients over the age of 18 who underwent abdominal SCP between 2019 and 2023 were included. The cohort was formed into two groups: (1) Patients who underwent SCP through a mini-laparotomy incision (Mini-lap group); (2) Patients who underwent laparoscopic or robotic-assisted laparoscopic SCP (Lap/Robot group).

**Results:**

A total of 116 patients were included in the final analysis. Ninety patients underwent either laparoscopic or robotic-assisted SCP, whereas 26 patients underwent SCP with a mini-laparotomy approach. Study participants exhibited a mean age of 63.1 ± 10.3 years, mean body mass index (BMI) of 25.8 ± 4.9 Kg/m^2^, and 77.6% of them identified as Caucasian. Upon comparison of demographic and past medical history between groups there were no statistically significant differences in age, BMI, menopausal status, race, parity or comorbid conditions. Patients in the Mini-lap group were less likely to have undergone previous abdominal surgery (11.5% vs. 50.6%, *p* < 0.001) and had more severe apical prolapse (stage 4 prolapse, 40% vs. 21.2%, *p* < 0.001) than their counterparts in the Lap/robot group. Regarding intraoperative parameters, length of surgery was significantly shorter in the Mini-lap group compared to the Lap/robot group (97.3 ± 35.0 min vs. 242.0 ± 52.6 min, *p* < 0.001). When focusing on the primary outcome, postoperative complications within the first 30 days after surgery, there were no differences noted between groups. Additionally, the number of unanticipated healthcare encounters, such as phone calls, clinic visits, emergency department visits, urgent care visits, readmissions and reoperations were similar between groups.

**Conclusions:**

Mini-laparotomy approach for SCP is safe with comparable intra- and postoperative complications, and unanticipated healthcare encounters compared to conventional minimally invasive methods.

## Introduction

Pelvic organ prolapse (POP) is defined as descent of one or more vaginal compartments, including the cervix, apex of the vagina (after a hysterectomy), anterior, or posterior vaginal walls [1–3]. While POP may be managed with conservative methods, such as pelvic floor physical therapy or pessary, many women interested in a definitive solution for their prolapse will opt for surgical treatment. Over the years, a wide array of surgical approaches has been described, including vaginal and abdominal (open, laparoscopic and robotic assisted laparoscopy), with native tissue repair or mesh reinforced suspension [3–8].

Abdominal sacrocolpopexy (SCP) is an established and effective surgical procedure for restoration of apical support [9–12]. Originally, this surgery was described using a laparotomy approach. However, this technique was associated with significant morbidity [13, 14]. In recent years, minimally invasive approaches for SCP, with the use of traditional laparoscopic and robotic-assisted laparoscopic surgery, have gained considerable popularity due to their association with lower complication rates, less blood loss, and shorter hospital stays. More recent data demonstrating similar surgical outcomes between laparotomy and laparoscopic approaches has established minimally invasive surgery as the gold standard when performing SCP [14–16].

Mini-laparotomy was first introduced for benign gynecologic surgeries in 1996, with several studies showing its high efficacy in the treatment of uterine myomas (fibroids) [17, 18]. This alternative approach, consisting of a 4–8 cm suprapubic incision, has demonstrated similar perioperative outcomes and complications to the laparoscopic approach with shorter procedure length when performing myomectomy [18–21]. Currently, data evaluating a mini-laparotomy approach in the field of urogynecology, particularly during SCP, is lacking.

The aim of this study was to compare short-term outcomes including postoperative complications and unanticipated healthcare encounters between patients who underwent SCP with a mini-laparotomy approach compared to laparoscopic and robotic-assisted laparoscopic approaches. We hypothesized that patients undergoing SCP via mini-laparotomy route would have similar surgical outcomes and postoperative complications compared to patients who underwent SCP using laparoscopic/robotic-assisted surgery.

## Methods

We performed a retrospective cohort study including patients treated for apical prolapse at a tertiary, university-affiliated urogynecology practice. Patients over the age of 18 with apical prolapse who were indicated for surgical repair and underwent abdominal SCP between 2019 and 2023 were included. We excluded patients who underwent any apical procedure other than SCP, and those for whom data on the primary outcome of the study were missing. The cohort was formed into two groups: (1) Patients who underwent SCP through a mini-laparotomy incision (Mini-lap group); (2) Patients who underwent laparoscopic or robotic-assisted laparoscopic SCP (Lap/Robot group). This study was approved by the Institutional Review Board of NorthShore University Health System (IRB# EH22-132).

According to our division’s clinical routine, SCP is offered to patients with more severe prolapse, patients who are younger, more active, and those with prolapse recurrence following native tissue repair. Mini-laparotomy approach during SCP is performed by one of our senior attendings and is offered to patients planned for SCP as long as their BMI is under 35 Kg/m^2^. Patients were counseled regarding risks and benefits of SCP including differences between the proposed surgical approaches.

Patients’ electronic medical records were reviewed, and pre-, intra-, and postoperative data were collected systematically. Data points retrieved included demographics, prior medical and surgical history, symptom evaluation using the Pelvic Floor Distress Inventory (PFDI-20) questionnaire and Baden-Walker Halfway scoring system. PFDI-20 is a standardized questionnaire encompassing three subcategories which quantifies the severity of pelvic floor symptoms. These three subcategories include the Urinary Distress Inventory (UDI-6), the Pelvic Organ Prolapse Distress Inventory (POPDI-6), and the Colorectal-Anal Distress Inventory (CRADI-8) which assess for urinary incontinence, pelvic organ prolapse, and colorectal-anal distress, respectively [22]. The Baden-Walker Halfway scoring system is a clinical tool which categorizes the descent of pelvic organs with respect to the hymenal ring and ranges from zero to 4, four being the complete protrusion of the prolapsed organ [23]. Operative and postoperative notes were reviewed, and data on intra- and perioperative characteristics were compiled. Electronic medical records were carefully surveyed to identify unanticipated healthcare encounters, emergency department or urgent care visits, hospital readmissions, and reoperations.

The primary outcome of the study was a comparison of complications within the first 30 days after surgery between women who underwent SCP with a mini-laparotomy approach compared to women who underwent laparoscopic and robotic-assisted laparoscopic SCP. Secondary outcomes included a comparison of unanticipated healthcare encounters between groups.

### Surgical technique

**Fig. 1:**
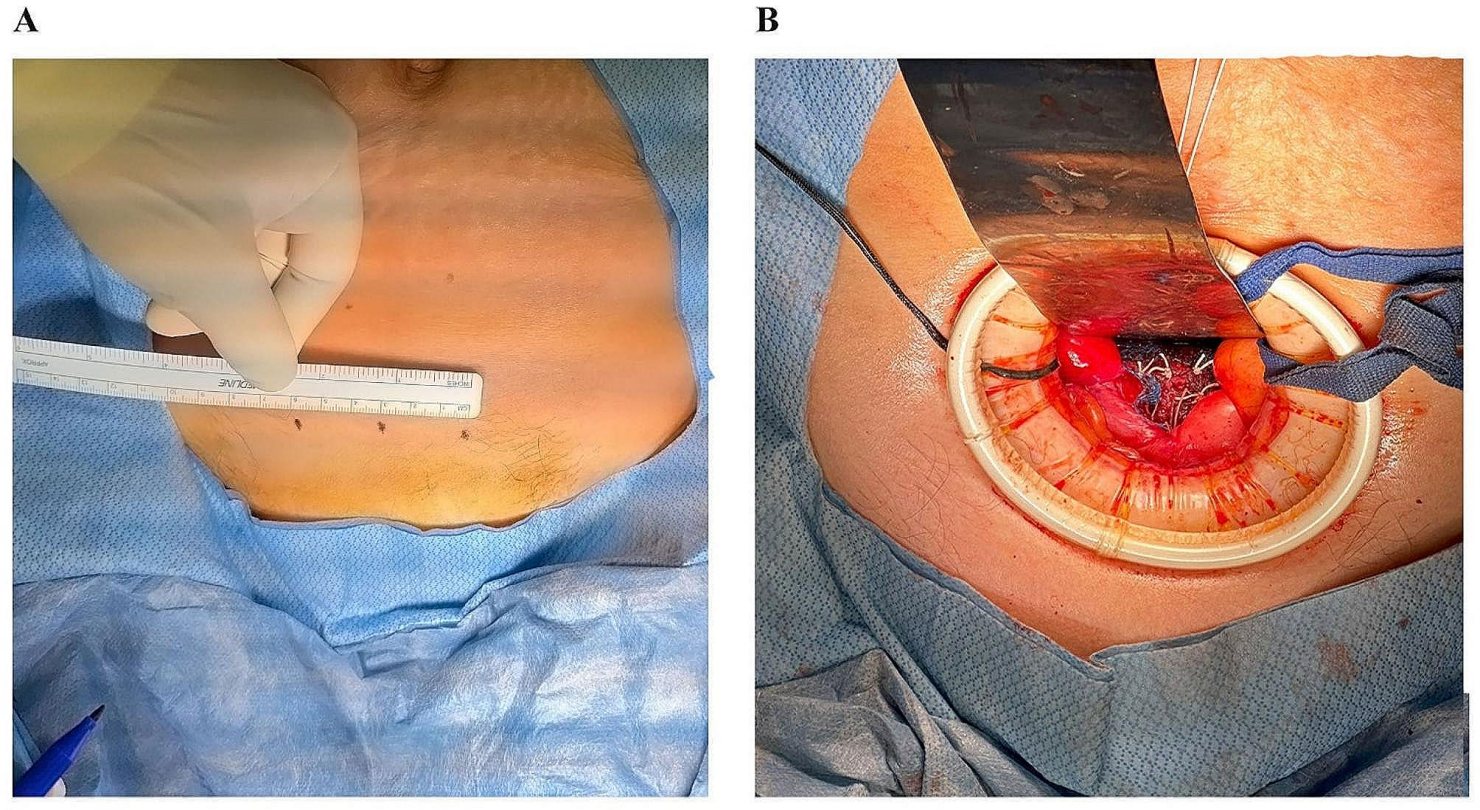
(**A**) Marking of a mini-laparotomy incision. (**B**) Mesh is sutured to the vaginal apex and anterior vaginal wall. The Alexis Wound Protector/Retractor is used to achieve optimal visualization

All procedures were performed under general anesthesia by four board-certified female pelvic medicine and reconstructive surgeons all of which were well trained in performing SCP. A first-generation cephalosporin was administered for surgical prophylaxis. For the Mini-lap group, a 5–6 cm horizontal incision was made approximately two cm superior to the pubic symphysis (Fig. 1), followed by dissection down to the rectus fascia. The fascia was incised vertically, and the peritoneum was identified and entered bluntly. The bowel was packed away with moist laparotomy sponges, and an Alexis® retractor (Applied Medical, Rancho Santa Margarita, CA) was placed within the abdomen. This retractor enables for the small incision to be shifted using retractors thus providing relevant exposure for each stage of the surgery. At this point, a supracervical hysterectomy was performed in standard fashion, if the patient still had a uterus.

The peritoneal tissue on the anterior (vesicovaginal) aspect of the vaginal cuff was incised using Metzenbaum scissors, and the vesicovaginal space was dissected, thereby exposing the vagina and mobilizing the bladder safely away. An identical technique was used posteriorly in the rectovaginal space, exposing the posterior vaginal surface. The Upsylon Y-Mesh™ (Boston Scientific Corporation, Natick, MA) was then fastened to the vagina with the anterior leaf of mesh sutured to the anterior vaginal surface, utilizing a combination of 0-vicryl and CV-2 Gore-Tex sutures (Fig. 1). An identical technique was used to affix the posterior mesh leaf to the posterior vagina. Moist laparotomy sponges were used to pack the small bowel contents superiorly and the sigmoid toward the left side. This resulted in exposure of the sacral promontory. The surrounding key landmarks were identified, and the peritoneum overlying the promontory was incised using the Metzenbaum scissors. This incision was extended toward the vagina with careful attention to the rectum medially and ureter and sidewall laterally. Two CV-2 Gore-Tex sutures were placed into the anterior longitudinal ligament overlying the sacrum at approximately the S2/S3 level. These sutures were then fastened to the sacral tail of the mesh and tied down. Tensioning was then checked both abdominally, and with palpation of the vagina to confirm proper elevation without excessive tension. At this point closure of the posterior peritoneum was performed, eventually incorporating the vesical peritoneum, resulting in complete reperitonealization of the mesh. Hemostasis was confirmed throughout the surgical field, and all abdominal packs and the ring retractor were removed. The fascia was closed followed by subcutaneous skin closure. Concomitant procedures such as anterior and posterior colporrhaphy and mid-urethral sling procedures were performed as indicated. Blood loss during the procedure was estimated by the primary surgeon.

### Statistical analyses

Patient characteristics, intraoperative factors, postoperative complications and unanticipated healthcare encounters 30 days after surgery were compared between two groups using Chi-squared test or Fisher’s exact test for categorical variables and Student t-test (parametric) or Mann-Whitney U test (nonparametric) for continuous variables. The normal distribution of continuous variables was assessed using the Kolmogorov-Smirnov test. Statistical analyses were performed using SAS 9.4 (SAS Institute, Cary, NC), and a p-value < 0.05 was considered statistically significant.

## Results

A total of 116 patients were included in the final analysis. Ninety patients underwent either laparoscopic or robotic-assisted SCP, whereas 26 patients underwent SCP with a mini-laparotomy approach. The study participants exhibited a mean age of 63.1 ± 10.3 years, mean body mass index (BMI) of 25.8 ± 4.9 Kg/m^2^, and 77.6% of them identified as Caucasian.

Table 1 includes a comparison of the main demographic and preoperative variables. There were no statistically significant differences observed between the two groups in terms of age, BMI, menopausal status, race, parity or comorbid conditions. Patients in the Lap/Robot group had a higher prevalence of previous abdominal surgery compared to the Mini-Lap group. No other differences were noted regarding prior surgical history.

**Table 1:** Demographic and preoperative characteristics of patients with mini-laparotomy vs. laparoscopic/robotic approach

	Laparoscopic/robotic (*N* = 90)		Mini-laparotomy (*N* = 26)		p-value
	n	%	n	%	
Age at time of surgery, mean ± SD	90	62.9 ± 10.4	26	64.0 ± 10.0	0.615
Parity, median (range)	75	2 (1–7)	24	2 (0–6)	0.436
**Race**					
Caucasian	71	78.9	19	73.1	0.270
Asian	2	2.2	2	7.7	
Other	16	17.8	4	15.4	
Declined/Unknown	1	1.1	1	3.8	
**Ethnicity**					
Non-hispanic	82	91.1	25	96.1	0.197
Hispanic	7	7.8	0	0.0	
Declined/Unknown	1	1.1	1	3.8	
**Smoking status**					
Never	55	67.9	17	70.8	1.000
Former	25	30.9	7	29.2	
Current	1	1.2	0	0.0	
**Menopausal status**					
Pre	12	14.6	2	7.7	0.511
Post	70	85.4	24	92.3	
BMI, mean ± SD	90	26.1 ± 5.0	26	25.0 ± 4.5	0.329
**Comorbid conditions**					
IDDM	2	2.3	1	3.8	0.544
DM	5	5.7	3	11.5	0.380
HTN	25	28.4	4	15.4	0.180
Cardiovascular disease	12	13.6	2	7.7	0.518
Respiratory disease	5	5.7	3	11.5	0.380
Depression/Anxiety	15	17.0	5	19.2	0.775
**Surgical history**					
Prior hysterectomy	19	21.1	3	11.5	0.396
Prior abdominal surgery	42	50.6	3	11.5	< 0.001
Prior prolapse surgery	11	13.1	0	0.00	0.063
Prior SUI surgery	9	10.7	0	0.00	0.112
**Baden-Walker and PFDI-20 measurements**					
**Anterior vaginal wall prolapse (Cystocele)**					
1	0	0.0	3	12.0	< 0.001
2	26	30.9	3	12.0	
3	37	44.0	5	20.0	
4	21	25.0	14	56.0	
**Posterior vaginal wall prolapse (Rectocele)**					
1	1	1.2	11	44.0	< 0.001
2	68	80.9	3	12.0	
3	7	8.3	3	12.0	
4	8	9.5	8	32.0	
**Uterine/Vault/Cervix prolapse**					
1	2	7.1	6	24.0	< 0.001
2	39	45.9	6	24.0	
3	22	25.9	3	12.0	
4	18	21.2	10	40.0	
**Prolapse stage**					
1	0	0.0	3	12.0	< 0.001
2	32	37.2	2	8.0	
3	35	40.7	5	20.0	
4	19	22.1	15	60.0	
PFDI-20 (0-300)	59	87.3 ± 51.9	23	91.5 ± 58.0	0.749
POPDI-6 (0-100)	59	37.3 ± 21.0	23	36.0 ± 23.5	0.810
CRADI-8 (0-100)	59	17.5 ± 18.1	23	21.6 ± 19.9	0.376
UDI-6 (0-100)	58	33.0 ± 21.4	23	33.9 ± 26.6	0.877
Preoperative hemoglobin	79	13.3 ± 1.0	24	13.3 ± 1.6	0.978

All data are presented as mean ± SD, median (range) or n (%)

*Note* BMI, body mass index; IDDM, insulin-dependent diabetes mellitus; DM, diabetes mellitus; HTN, hypertension; SUI, stress urinary incontinence; PFDI-20, Pelvic Floor Disability Index; POPDI, Pelvic Organ Prolapse Distress Inventory; CRADI, Colorectal-Anal Distress Inventory; UDI, Urinary Distress Inventory

Preoperative PFDI-20 scores were similar between groups. Analysis of prolapse stage demonstrated a significant difference between the two cohorts. Specifically, the majority of patients in the Mini-Lap group had stage 4 uterine prolapse compared to stage 2 in the Lap/Robot group. Furthermore, patients in the Mini-Lap group had increased cystocele stage compared to their Lap/Robot group counterparts.

Intraoperative variables are tabulated in Table 2. All patients underwent general anesthesia. No differences were noted between groups regarding American Society of Anesthesia Score (ASA), and concomitant anti-incontinence procedure. Operative time was significantly shorter in the Mini-Lap group compared to Lap/Robot group (97.3 ± 35.0 min vs. 242.0 ± 52.6, *p* < 0.001). Patients in the Mini-Lap group had a higher rate of concomitant vaginal repair, with anterior and posterior colporrhaphy. No statistically significant differences were found between groups regarding intraoperative complications.

**Table 2:** Intraoperative characteristics of patients with mini-laparotomy vs. laparoscopic/robotic approach

	Laparoscopic/robotic (*N* = 90)		Mini-laparotomy (*N* = 26)		p-value
	n	%	n	%	
**ASA grade**					
1	17	19.3	4	15.4	0.408
2	61	69.3	16	61.5	
3	9	10.2	6	23.1	
4	1	1.1	0	0.0	
Concomitant procedure*					
RMUS	41	45.6	11	42.3	0.700
Anterior repair	3	3.3	10	38.5	< 0.001
Posterior repair	8	8.9	11	42.3	< 0.001
Revision of prior MUS	6	2.9	3	4.3	0.696
Rectopexy	3	3.3	1	3.8	1.000
Perineorrhaphy	5	5.6	4	15.4	0.112
Intraoperative complications*					
None	88	98.9	26	100.0	1.000
Cystostomy	1	1.1	0	0.00	
Procedure length (minutes)	89	242.0 ± 52.6	25	97.3 ± 35.0	< 0.001
EBL (mL)	85	84.5 ± 68.4	26	63.6 ± 58.9	0.163

All data are presented as mean ± SD, median (range) or n (%)

*May have multiple responses so percentages do not add up to 100%

*Note* ASA, American Society of Anesthesiology; RMUS, retropubic midurethral sling; MUS, midurethral sling; EBL, estimated blood loss

Postoperative variables are presented in Table 3. All patients were discharged on the day of surgery or postoperative day one. There was no difference between groups regarding the ability to achieve same day discharge (53.4% vs. 61.5% in the Lap/robot and Mini-lap groups, respectively, *p* = 0.348). For the primary outcome, postoperative complications within the first 30 days after surgery, there were no differences noted between groups. Additionally, the number of unanticipated healthcare encounters, such as phone calls, clinic visits, emergency department visits, urgent care visits, readmissions or reoperations were similar between groups. Only one patient in the robotic/laparoscopic group required reoperation within 30 days, and this was due to a port site hernia.

**Table 3:** Postoperative parameters of patients with mini-laparotomy vs. laparoscopic/robotic approach

	Laparoscopic/robotic (*N* = 90)		Mini-laparotomy (*N* = 26)		p-value
	n	%	n	%	
**Complications during hospital admission**					
None	77	85.6	25	96.1	0.187
Nausea/vomiting	7	7.8	1	3.8	0.681
Pain	1	1.1	0	0.0	1.000
Hypotension	1	1.1	0	0.0	1.000
Desaturation	3	3.3	0	0.0	1.000
Dizziness	5	5.6	0	0.0	0.586
Immediate postoperative urinary retention	12	13.3	2	7.7	0.732
Discharge on day of surgery	62	53.4	16	61.5	0.348
Unanticipated healthcare encounters within 30 days of surgery*					
Clinic	8	8.9	5	19.2	0.163
ED	7	7.8	1	3.8	0.681
Phone calls	24	26.7	3	11.5	0.108
Reoperation	1	1.11	0	0.00	1.0000
Urgent care	1	0.5	1	1.4	0.442
**Complications within 30 days of surgery**					
None	87	96.7	26	100.0	1.000
UTI	2	2.2	0	0.0	
Reoperation due to port site hernia	1	1.1	0	0.0	

All data are presented as mean ± SD or n (%)

*Note* ED, emergency department; UTI, urinary tract infection

## Discussion

In this study, we present data showing similar intra- and postoperative complications in patients undergoing SCP using mini-laparotomy approach compared to patients who underwent laparoscopic or robotic-assisted laparoscopic SCP. We further found comparable rates of unanticipated healthcare encounters between groups. Procedure times for the mini-laparotomy approach were significantly shorter by over 2 h on average compared to laparoscopic/robotic surgery.

To date, SCP is considered the most durable surgery available for treatment of apical prolapse [19, 24, 25]. In recent years minimally invasive techniques such as straight stick and robotic-assisted laparoscopic surgery have emerged as the standard of care when performing SCP with low complication rates, less blood loss, and shorter hospital stay while maintaining high cure rates and low recurrence rates [26]. However, these approaches entail longer surgical time and higher costs [27, 28]. In this study, we propose SCP using mini-laparotomy approach as a minimally invasive alternative to laparoscopic/robotic surgery with shorter procedure length and similar short-term outcomes.

Previous studies focusing on comparison of mini-laparotomy and laparoscopic/robotic approaches in benign gynecologic surgery have been equivocal. Sirisabya et al. reported that performing a mini-laparotomy approach for hysterectomy might lead to similar outcomes with shorter surgical time [29]. In contrast, a large retrospective study including 680 patients who underwent myomectomy using either mini-laparotomy or laparoscopic approaches showed that patients in the laparoscopy group had less blood loss and shorter hospital stays. While surgical time was longer in the laparoscopy group, this difference was not statistically significant [30]. Supporting these findings, results of a recent metanalysis have shown that laparoscopic myomectomy is associated with shorter hospital stay, less blood loss, and fewer postoperative complications, such as ileus and fever, than mini-laparotomy [19]. Our findings demonstrate favorable short-term outcomes when utilizing mini-laparotomy during SCP, with comparable complications and unanticipated healthcare encounters compared to laparoscopic/robotic surgery. These differences may be explained by the fact that many of the studies comparing mini-laparotomy and laparoscopic/robotic approaches were performed in patients undergoing myomectomy; while both are benign gynecologic procedures, myomectomy usually entails higher blood loss and longer recovery times compared to SCP. Future larger studies comparing mini-laparotomy SCP and laparoscopic/robotic SCP are needed to confirm our findings.

Results of this study point towards mini-laparotomy as an alternative approach to other minimally invasive techniques during SCP. In our experience the smaller incision performed during mini-laparotomy, circumvents complications seen following the more traditional laparotomy approach. Furthermore, while we did not measure patient pain directly, patients’ ability to be discharged on the same day of surgery may be looked at as a proxy for pain control and did not differ between groups. While these results reinforce mini-laparotomy as a minimally invasive technique, long-term outcomes are paramount in evaluating its safety and efficacy.

The primary areas of concern regarding emerging minimally invasive surgical techniques, such as laparoscopic and robotic-assisted surgeries, have been associated costs and increased duration of surgical procedures. We found that surgical times were substantially shorter, by over two hours, when performing mini-laparotomy compared to laparoscopic/robotic SCP. Apart from shortening anesthetic times for all patients, this may benefit patients with comorbidities who are anticipated to experience worse outcomes should the surgical procedure be prolonged [16, 31, 32] or require steep Trendelenberg positioning. Furthermore, as the mini-laparotomy procedure results in reduced utilization of medical resources, such as operating room time and equipment, it would save money for both patients and healthcare facilities.

Performing SCP using mini-laparotomy poses certain challenges to the surgical team. Patient obesity may limit access to the sacrum and visibility of the posterior vaginal wall. Furthermore, obese patients may experience higher rates of surgical site infection. While the size of our cohort did not allow for subgroup analysis according to BMI, we believe that patient selection is key to the success of this approach.

Strengths of the study include its comparative design and ability to address a relevant clinical question for which there is currently limited data. To our knowledge, this is one of the only studies to report on a mini-laparotomy approach during SCP using comparative methodology. Lastly, we were able to report on unanticipated healthcare encounters, including phone calls, clinic visits, emergency department and urgent care visits thanks to the detailed documentation within the NorthShore University HealthSystem database.

This study has certain limitations including its retrospective nature, lack of formal power analysis and relatively small sample size. Due to the latter, we were unable to conduct subgroup analyses within our cohort. Data on postoperative pain assessment was unavailable for most patients. Furthermore, we were unable to report on the surgical success of the procedures performed. However, previous studies comparing laparotomy and minimally invasive approaches during SCP found similar surgical outcomes or more favorable outcomes with an open approach. Future studies should aim to be larger with longer follow-up duration. This will allow detection of complications which are less prevalent and to evaluate surgical outcomes according to surgical approach.

In conclusion, our study shows that the mini-laparotomy approach to SCP is safe with comparable intra- and postoperative complications and unanticipated healthcare encounters compared to conventional minimally invasive methods. Importantly, we found that operative times for this procedure were significantly shorter compared to laparoscopic/robotic surgery. Clinicians may find this approach advantageous in certain clinical scenarios. Future studies are needed to confirm our findings on a larger scale and to better define surgical efficacy of using mini-laparotomy for SCP.

## Data Availability

No datasets were generated or analysed during the current study.
